# Soma size distinguishes projection neurons from neurokinin 1 receptor-expressing interneurons in lamina I of the rat lumbar spinal dorsal horn

**DOI:** 10.1016/j.neuroscience.2009.09.071

**Published:** 2009-12-29

**Authors:** K.S. Al Ghamdi, E. Polgár, A.J. Todd

**Affiliations:** Neuroscience and Molecular Pharmacology, Faculty of Biomedical and Life Sciences, West Medical Building, University Avenue, University of Glasgow, Glasgow G12 8QQ, UK

**Keywords:** spinoparabrachial, caudal ventrolateral medulla, lateral parabrachial area, retrograde tracing, interneuron, pyramidal cell, CTb, cholera toxin B subunit, CVLM, caudal ventrolateral medulla, LPb, lateral parabrachial area, NK1r, neurokinin 1 receptor, PAG, periaqueductal grey matter

## Abstract

Lamina I of the spinal dorsal horn contains neurons that project to various brain regions, and ∼80% of these projection cells express the neurokinin 1 receptor (NK1r), the main receptor for substance P. Two populations of NK1r-immunoreactive neurons have been identified in lamina I: small weakly immunoreactive cells and large cells with strong immunolabelling [Cheunsuang O and Morris R (2000) Neuroscience 97:335–345]. The main aim of this study was to test the hypothesis that the large cells are projection neurons and that the small cells are interneurons. Projection neurons were identified by injection of tracers into the caudal ventrolateral medulla and lateral parabrachial area, and this was combined with immunostaining for NK1r. We found a bimodal size distribution for NK1r-immunoreactive neurons. The small cells (with somatic cross-sectional areas <200 μm^2^) showed weak immunoreactivity, while immunostaining intensity was variable among the large cells. Virtually all (99%) of the immunoreactive cells with soma areas >200 μm^2^ were retrogradely labelled, while only 10% of retrogradely labelled cells were smaller than this. Soma sizes of retrogradely labelled neurons that lacked NK1r did not differ from those of NK1r-expressing projection neurons. It has been suggested that a population of small pyramidal projection neurons that lack NK1r may correspond to cells activated by innocuous cooling, and we therefore assessed the morphology of retrogradely labelled cells that were not NK1r-immunoreactive. Fifteen percent of these were pyramidal, but these did not differ in size from pyramidal NK1r-immunoreactive projection neurons. These results confirm that large NK1r-immunoreactive lamina I neurons are projection cells, and suggest that the small cells are interneurons. Since almost all of the NK1r-immunoreactive cells with soma size >200 μm^2^ were retrogradely labelled, cells of this type can be identified as projection cells in anatomical studies.

Lamina I of the dorsal horn (Rexed, 1952) is innervated by nociceptive and thermoreceptive primary afferents (Light and Perl, 1979; Sugiura et al., 1986) and contains neurons activated by noxious and/or thermal stimuli (Christensen and Perl, 1970; Han et al., 1998; Zhang et al., 2006). Although many neurons in lamina I have axons that remain in the spinal cord, it also contains cells that project to the brain (Willis and Coggeshall, 2004; Craig, 1995; Villanueva and Bernard, 1999). In the rat, supraspinal targets for these cells include the caudal ventrolateral medulla (CVLM), the nucleus of the solitary tract, lateral parabrachial area (LPb), periaqueductal grey matter (PAG) and thalamus (Menétrey et al., 1982, 1983; Cechetto et al., 1985; Hylden et al. 1989; Lima and Coimbra, 1988, 1989; Burstein et al., 1990; Lima et al., 1991; Todd et al., 2000; Spike et al., 2003; Al-Khater et al., 2008). These projections are mainly contralateral, with a smaller bilateral component (Spike et al., 2003), and many lamina I neurons can be retrogradely labelled from more than one brain region (Hylden et al., 1989; Spike et al., 2003; Al-Khater and Todd, 2009).

Many nociceptive primary afferents contain substance P (Hökfelt et al., 1975; Lawson et al., 1997), which is released from their central terminals following noxious stimulation (Duggan et al., 1987; Mantyh et al., 1995). Substance P acts on neurokinin 1 receptors (NK1rs), which are present at high density in lamina I (Bleazard et al., 1994; Liu et al., 1994; Nakaya et al., 1994; Brown et al., 1995; Littlewood et al., 1995). NK1r-expressing dorsal horn neurons have attracted particular interest because they are activated by noxious stimuli (Henry, 1976; Doyle and Hunt, 1999), and also because ablation of these cells with substance P conjugated to saporin causes a dramatic reduction of hyperalgesia in inflammatory and neuropathic pain models (Mantyh et al., 1997; Nichols et al., 1999).

Several studies have demonstrated that the NK1r is expressed by lamina I projection neurons (Li et al., 1996, 1998; Todd, 2002; Yu et al., 2005; Almarestani et al., 2007, 2009), and we have found that the receptor is present on approximately 80% of lamina I neurons that are retrogradely labelled from thalamus, PAG, LPb or CVLM (Marshall et al., 1996; Todd et al., 2000; Al-Khater et al., 2008). However, although ∼45% of lamina I neurons are NK1r-immunoreactive (Todd et al., 1998), it has been reported that projection cells make up only 5–10% of the neuronal population in this lamina (Bice and Beal, 1997a, 1997b; Spike et al., 2003; Al-Khater et al., 2008) and the receptor must therefore be present on many lamina I interneurons.

Cheunsuang and Morris (2000) identified two distinct populations of NK1r-expressing neurons in lamina I: small cells that stained weakly for the receptor and large cells that were described as strongly immunoreactive. We subsequently reported that a sample of 45 NK1r-immunoreactive lamina I neurons retrogradely labelled from CVLM had somata that were similar in size to those of the large cells identified by Cheunsuang and Morris (Polgár et al., 2002). However, we could not determine whether all of the large cells were projection neurons, since not all projection neurons in this lamina would have been labelled from the CVLM (Spike et al., 2003). The main aim of the present study was to test the hypothesis that all of the large lamina I NK1r-immunoreactive cells identified by Cheunsuang and Morris are projection neurons, while the small cells are interneurons. To achieve this, we injected tracers into both CVLM and LPb, since it has been shown that virtually all lamina I projection neurons can be labelled from these sites (Spike et al., 2003).

Several studies have examined morphology of lamina I neurons in various species (Lima and Coimbra, 1983, 1986; Zhang et al., 1996; Zhang and Craig, 1997; Han et al., 1998; Yu et al., 1999; Spike et al., 2003; Almarestani et al., 2007, 2009), and have described three major types: fusiform, pyramidal and multipolar. It has been reported that pyramidal cells are selectively activated by innocuous cooling, while cells belonging to the other classes respond to noxious stimuli (Han et al., 1998). However, we found that the majority of pyramidal projection neurons were NK1r-immunoreactive (Spike et al., 2003; Al-Khater and Todd, 2009). Recent reports have described a distinctive population of small pyramidal lamina I projection neurons that lacked the NK1r (Almarestani et al., 2007, 2009), and we therefore examined the morphology of retrogradely labelled neurons that were not NK1r-immunoreactive, in order to identify cells of this type.

## Experimental procedures

### Animals and operative procedures

All experiments were approved by the Ethical Review Process Applications Panel of the University of Glasgow and were performed in accordance with the European Community directive 86/609/EC and the UK Animals (Scientific Procedures) Act 1986. All efforts were made to minimize the number of animals used and their suffering.

Three adult male Wistar rats (250–280 g; Harlan, Loughborough, UK) were anaesthetized with isofluorane and placed in a stereotaxic frame, after which anaesthetic was administered through a mask attached to the frame. Each rat received two injections: (1) 50 nl of 4% Fluorogold (Fluorochrome Inc, Englewood, CO, USA) targeted on the left lateral parabrachial area, and (2) 200 nl of 1% cholera toxin B subunit (CTb; Sigma-Aldrich, Poole, UK) into the left CVLM. All injections were made through glass micropipettes, and in each case a different pipette was used for each tracer. The animals made an uneventful recovery from anaesthesia. After a 3 day survival period they were re-anaesthetized with pentobarbitone (300 mg i.p.) and perfused through the heart with a fixative that contained 4% freshly de-polymerized formaldehyde. The brain and lumbar spinal cord were dissected out and post-fixed for at least 4 h. The brain was cryoprotected in 30% sucrose overnight.

### Tissue processing and immunocytochemistry

The regions of the brainstem that contained the injection sites were cut into 100 μm thick coronal sections with a freezing microtome. Sections through the Fluorogold injection were mounted in anti-fade medium and viewed with epi-fluorescent illumination and an UV filter set. Sections through the CTb injection were reacted with goat anti-CTb (List Biological Laboratories, Campbell, CA, USA; diluted 1:50,000) using an immunoperoxidase method as described previously (Todd et al., 2000). In all cases the spread of tracer from the injection sites was plotted onto drawings of the brainstem (Paxinos and Watson, 2005), and representative examples were photographed.

The L4 segments from each animal were notched on the left side (ipsilateral to the injections) and cut into 60 μm horizontal sections with a Vibratome. These were incubated free-floating at 4 °C for 3 days in a cocktail consisting of guinea-pig anti-Fluorogold (Protos Biotech Corp., New York, USA, 1:500), goat anti-CTb (1:5000) and rabbit anti-NK1r (Sigma-Aldrich, 1:10,000). They were then reacted with species-specific secondary antibodies raised in donkey conjugated to either Alexa 488 (Invitrogen, Paisley, UK; 1:500), or to Rhodamine Red or Cy5 (Jackson Immunoresearch, West Grove, PA, USA; 1:100). The sections were mounted in serial order in anti-fade medium and stored at −20 °C. All antibodies were diluted in phosphate-buffered saline that contained 0.3% Triton-X100 (to enhance antibody penetration) and 0.3 M NaCl. We have found that the relatively high concentration of NaCl in this buffer reduces the non-specific binding of antibodies, and avoids the need for addition of other proteins, such as blocking sera.

The NK1r antibody (catalogue number S8305) was raised in rabbit against amino acids 393-407 of the rat NK1r conjugated to keyhole limpet haemocyanin. It has been shown that there is no immunostaining with this antibody in sections of medulla and cervical spinal cord from mice in which the NK1r has been deleted (NK1^−/−^), while staining is present in sections from wild-type mice (Ptak et al., 2002). Specificity of the CTb and Fluorogold antibodies was shown by the lack of staining in regions of the CNS that did not contain neurons that had transported the tracer, and by the presence of immunostaining in populations of neurons that are known to project to the injection sites.

### Confocal microscopy and analysis

Sections from the L4 spinal segment were initially examined with fluorescence microscopy, and those on the right (un-notched) side that contained lamina I were identified by the presence of numerous retrogradely labelled cells. In this way, between one and three sections from each animal were selected for further analysis. These sections were scanned with a confocal microscope (Bio-Rad Radiance 2100; Bio-Rad, Hemel Hempstead, UK) through a 40× oil-immersion lens to produce image stacks with a 2 μm z-separation. Because the area covered by this lens was approximately 300×300 μm^2^, several overlapping fields (between 11 and 15 from each animal) were scanned in order to include most of the medial two-thirds of lamina I. The lateral part of the dorsal horn was not analysed, as the orientation of the lamina is different in this region. Scans were performed sequentially with each laser line to avoid fluorescent bleedthrough.

Confocal image stacks were analysed with Neurolucida for Confocal software (MicroBrightField Inc., Colchester, VT, USA). Initially, only the channel corresponding to NK1r-immunoreactivity was viewed and this was used to identify lamina I, which has a relatively high density of NK1r-immunoreactivity compared to lamina II. NK1r-immunoreactive cells in lamina I were then selected and the outlines of their cell bodies were drawn by examining all of the optical sections through each cell. Preliminary observation confirmed that there was a large population of small cells (<200 μm^2^ soma cross-sectional area) that showed weak NK1r-immunoreactivity, and these were found to be far more numerous than the larger NK1r-immunoreactive cells. For this reason, all of the larger cells (>∼200 μm^2^ soma cross-sectional area) together with a representative sample of small cells were selected for analysis. For each of the selected cells, the maximum cross-sectional area of the soma was measured from the drawings (Puskár et al., 2001). In addition, the intensity of NK1r-immunostaining in the plasma membrane was assigned a score ranging from 4 (strong) to 1 (very weak), as described previously (Al-Khater and Todd, 2009). This scoring system was used because variation in immunofluorescence intensity at different depths of the Vibratome sections makes it difficult to use a more objective measure (Spike et al., 2003). When all of the selected cells in a field had been analysed, the channels corresponding to Fluorogold and CTb were examined, and the presence or absence of these tracers in each of the selected cells was determined. In addition, any retrogradely-labelled cells that had not been included in the selected sample were identified and analysed in the same way. These additional cells included those that were NK1r-negative as well as some that showed very weak immunostaining and had not been recognized in the initial survey. Cells were excluded from the analysis if part of the soma had been removed from the Vibratome section in such a way that the cross-sectional area would be underestimated.

Since it has been reported that there is a distinctive population of pyramidal spinoparabrachial neurons that lack the NK1r, we analysed the morphology of all retrogradely labelled cells that were not NK1r-immunoreactive, by examining confocal image stacks with Neurolucida software. The criteria described by Zhang et al. (1996) and Zhang and Craig (1997) were used to assign cells to multipolar, pyramidal or fusiform classes. Attention was paid to the soma size and the number and orientation of primary dendrites, as it has been suggested that these features are particularly important for classification (Almarestani et al., 2009).

## Results

### Injection sites

**Fig. 1:**
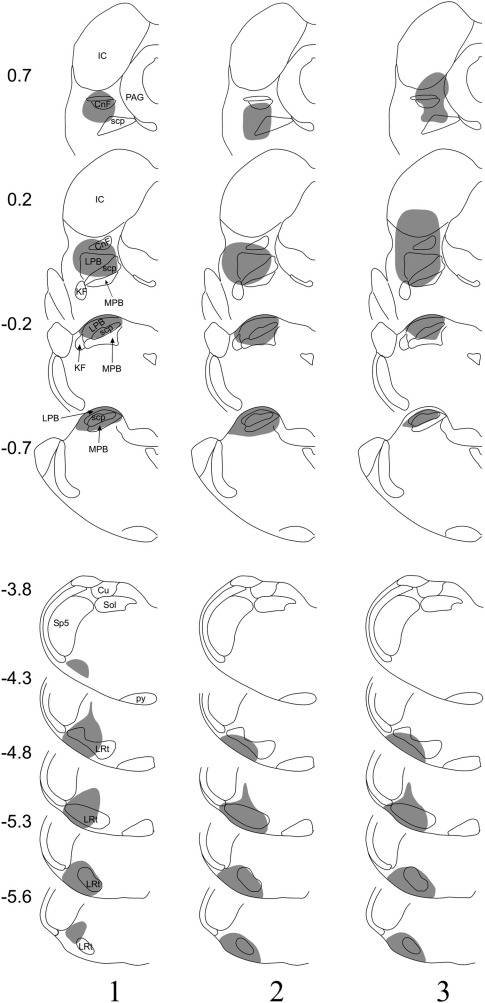
Fluorogold and CTb injection sites in the three experiments. The drawings show the spread of tracer in each of these experiments. Each vertical column represents a single experiment, and the experiment number is shown at the bottom of the column. Numbers on the left give the approximate antero-posterior positions of the sections relative to the interaural plane. Drawings are based on those of Paxinos and Watson (2005). The upper four outlines in each column represent the Fluorogold injection (targeted on the LPb), while the lower five show the spread of CTb (injected into the CVLM). CnF, cuneiform nucleus; Cu, cuneate nucleus; IC, inferior colliculus; KF, Kölliker-Fuse nucleus; LPB, lateral parabrachial nucleus; LRt, lateral reticular nucleus; MPB, medial parabrachial nucleus; PAG, periaqueductal grey matter; py, pyramidal tract; scp, superior cerebellar peduncle; Sol, nucleus of the solitary tract; Sp5, spinal trigeminal nucleus.

**Fig. 2:**
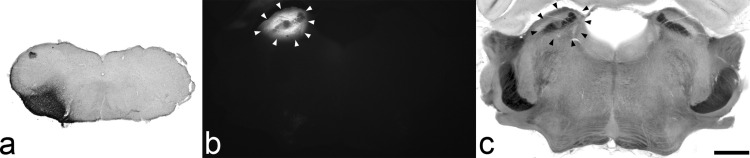
Examples of CTb and Fluorogold injection sites. (a): section (interaural ∼−5.3 mm) through the CVLM injection in experiment 2 which had been reacted with an immunoperoxidase method to reveal CTb. (b, c): fluorescent and brightfield photomicrographs of a section (interaural ∼−0.2 mm) through the Fluorogold injection in experiment 3. The spread of tracer is indicated by arrowheads. Scale bar=1 mm.

The extent of spread of tracer in each experiment is illustrated in Fig. 1 and photomicrographs of representative injection sites are shown in Fig. 2. The Fluorogold injection covered most or all of the LPb in each case, with variable spread into surrounding structures such as the superior cerebellar peduncle, medial parabrachial area, cuneiform nucleus and inferior colliculus. The CTb injections filled the lateral part of the lateral reticular nucleus in all three cases, with spread into surrounding regions, including the area between this nucleus and the spinal trigeminal nucleus and the ventrolateral white matter.

### NK1r-immunoreactivity in lamina I

**Fig. 3:**
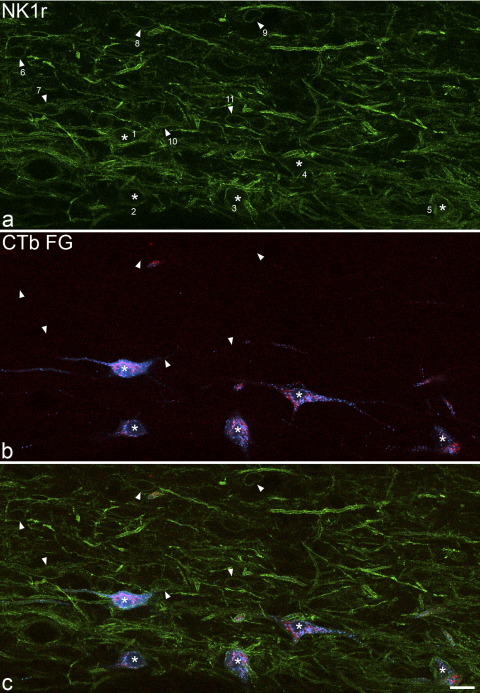
Immunoreactivity for NK1r and the two retrograde tracers in a horizontal section from the L4 segment of experiment 2. (a) shows a field scanned to reveal NK1r-immunoreactivity (green), (b) has been scanned for CTb (red) and Fluorogold (FG, blue), while (c) is a merged image. The NK1r-immunoreactivity is associated with thin elongated profiles, which are dendrites, as well as with larger structures, which are cell bodies. This field contains the cell bodies of five NK1r-immunoreactive projection neurons (asterisks, numbered 1–5) that were retrogradely labelled from both CVLM and LPb, and therefore contain both tracers. In addition, several smaller NK1r-immunoreactive cell bodies are visible, and these are not retrogradely labelled. Six of these cells are indicated with arrowheads (numbered 6–11). Cells 1 and 5 were assigned a NK1r-immunoreactivity score of 3, cells 2–4 and 6–7 had a score of 2 and cells 8–11 a score of 1. The images are projections of two optical sections at 2 μm z-separation. Scale bar=20 μm.

Lamina I was characterized by a high density of NK1r-immunoreactive profiles, which could be identified as cell bodies or dendrites (Fig. 3a). As described by Cheunsuang and Morris (2000) the NK1r-immunoreactive cell bodies appeared to have a bimodal size distribution. Many small, weakly immunoreactive neurons were present, and these generally had a fusiform appearance, giving rise to two primary dendrites (one from each pole). In addition, larger cells with diverse somatodendritic morphology (including fusiform, pyramidal and multipolar types) were also present and these showed much more variability in the strength of NK1r-immunoreactivity, which ranged from strong to very weak. Although we did not analyse the dorsoventral distribution systematically, we observed that the small cells were particularly numerous in the ventral part of lamina I (as reported by Cheunsuang and Morris).

### Retrograde labelling

**Fig. 4:**
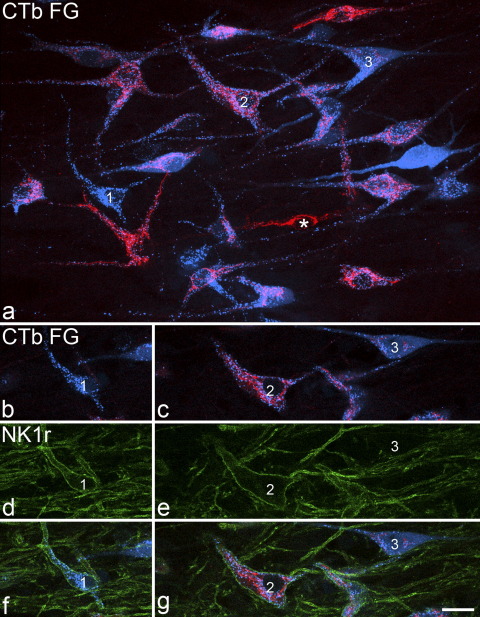
Examples of retrogradely labelled neurons in a horizontal section from L4 in experiment 1. (a) is a projected image through the cell bodies of several retrogradely labelled cells, with CTb shown in red and Fluorogold in blue. Most cells have taken up both tracers and therefore appear pink, while the cell numbered 1 is labelled only with Fluorogold, and the one marked with an asterisk is labelled only with CTb. The three numbered cells are pyramidal in shape and single optical sections through the cell bodies of each of these are shown in (b–g). From these images it can be seen that cells 1 and 2 are NK1r-immunoreactive, while cell 3 is not. Cell 1 was assigned a NK1r-immunoreactivity score of 3 and cell 2 was given a score of 4. The image in (a) is a projection of eight optical sections at 2 μm z-spacing. Scale bar=20 μm.

Altogether, 542 retrogradely labelled neurons were selected for analysis in lamina I in sections from the three experiments (156–206 per experiment). Of these, 83–88% contained both tracers, while 5–6% were labelled only with CTb and 5–12% were labelled only with Fluorogold (Table 1, Fig. 4a).

Between 76 and 86% (mean 81%) of the retrogradely labelled lamina I cells were NK1r-immunoreactive, and the strength of NK1r expression on these cells varied from very weak to strong (Table 2). Examples of retrogradely labelled neurons that were NK1r-immunoreactive or non-immunoreactive are shown in Figs. 3 and 4.

Of the 441 NK1r-immunoreactive retrogradely labelled neurons, 420 had been identified in the initial surveys of NK1r-immunoreactivity in the confocal image stacks (see Methods). The remaining 21 (most of which showed weak immunoreactivity) were identified only after the channels corresponding to CTb and Fluorogold were observed.

### Soma cross-sectional areas

**Fig. 5:**
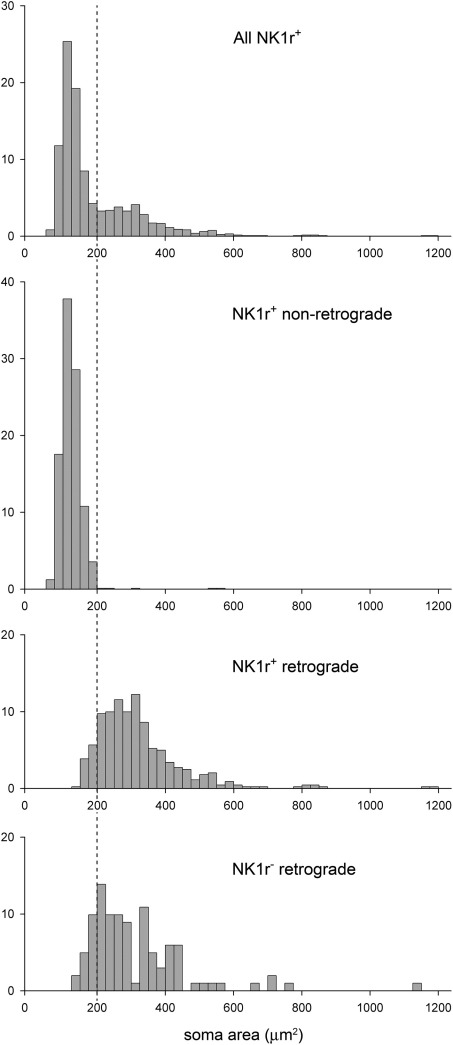
Frequency histograms showing the soma cross-sectional areas for different groups of lamina I neuron: all of the NK1r-immunoreactive ones (All NK1r^+^, *n*=1341), those that were NK1r-immunoreactive but not retrogradely labelled (NK1r^+^ non-retrograde, *n*=900), those that were NK1r-immunoreactive and retrogradely labelled (NK1r^+^ retrograde, *n*=441) and those that were retrogradely labelled and not NK1r-immunoreactive (NK1r^−^ retrograde, *n*=101). In each case, the y-axis represents percentage. The dashed line corresponds to a cross-sectional area of 200 μm^2^. The NK1r-immunoreactive neurons show a clear bimodal distribution, with the first and second peaks corresponding to the non-retrogradely labelled and the retrogradely labelled populations, respectively.

In addition to the 441 retrogradely labelled cells, the sample of NK1r-immunoreactive neurons that had been selected from lamina I included 900 cells that were not retrogradely labelled (214–390 from each experiment). The great majority of these cells (784, 87%) were assigned a score of 1 for NK1r-immunoreactivity, while 106 (12%) were given a score of 2, and 10 (1%) a score of 3. Frequency distributions of soma cross-sectional areas for the different groups of neurons classified by NK1r expression and retrograde labelling are shown in Fig. 5. The histogram for all NK1r-immunoreactive neurons (*n*=1341) shows a clear bimodal distribution with the first peak corresponding to values between 50 and 200 μm^2^, a second broader peak extending from 200 to 600 μm^2^ and a few cells with larger areas (up to 1200 μm^2^). The cross-sectional areas of cell bodies for the NK1r-immunoreactive cells that were not retrogradely labelled ranged from 61 to 568 μm^2^, with a median value of 124 μm^2^ (*n*=900), and these corresponded to the first peak of the combined NK1r-immunoreactive group. The corresponding values for the retrogradely labelled NK1r-immunoreactive cells were 128–1198 μm^2^, with a median of 298 μm^2^ (*n*=441), and these corresponded to the second peak of the combined group. Soma areas for the retrogradely labelled cells that were NK1r-negative ranged between 137 and 1129 μm^2^ with a median of 272 μm^2^ (*n*=101). Differences between these three groups were found to be significant (Kruskal–Wallis test, *P*<0.001). Post-hoc Mann–Whitney *U*-tests with sequential Bonferroni correction showed a highly significant difference between the soma sizes of the NK1r-immunoreactive neurons that were not retrogradely labelled and those of both groups of retrogradely labelled neurons (*P*<0.001). However, there was no significant difference between the retrogradely labelled neurons that were NK1r-immunoreactive and those that were not immunoreactive (*P*=0.16).

Nearly all (895/900, 99.4%) of the NK1r-immunoreactive cells that were not retrogradely labelled had soma cross-sectional areas that were less than 200 μm^2^. In contrast, only 43/441 (9.8%) of the retrogradely labelled NK1r-positive cells had cell bodies smaller than 200 μm^2^ (Fig. 5).

### Morphology of NK1r-negative projection neurons

**Fig. 6:**
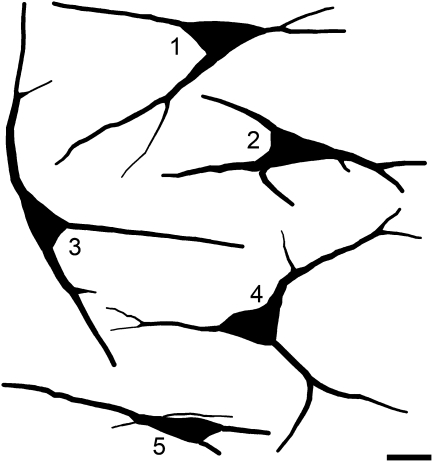
Drawings of five of the 15 retrogradely labelled pyramidal cells that were not NK1r-immunoreactive. Cell 1 corresponds to cell 3 in Fig. 4. Three of these cells (1, 3 and 4) each give rise to three primary dendrites. Cell 2 has two primary dendrites originating from one pole of the soma, while cell 5 has an additional very fine dendrite originating from the soma. Scale bar=20 μm.

Within the population of retrogradely labelled neurons that were not NK1r-immunoreactive (*n*=101), 15 were classified as pyramidal, 36 as multipolar and 24 as fusiform. Of the remainder, 13 had atypical features or were intermediate between two classes, while 13 could not be classified because of incomplete filling of primary dendrites. An example of a retrogradely labelled pyramidal cell that lacked NK1r-immunoreactivity is illustrated in Fig. 4 (cell 3) and Fig. 6 shows drawings of the cell bodies and proximal dendrites of five of these cells. The pyramidal cells were characterized by a triangular cell body, and in most cases (11/15) this gave rise to three primary dendrites (one from each pole of the soma). The remaining four pyramidal cells each had four primary dendrites. In one of these cases, two of the dendrites arose from one pole, while in the other three cases an additional thin dendrite was given off from the soma (Fig. 6). One of these travelled dorsally towards the dorsal columns. Although we did not analyse this in the present study, many of the retrogradely labelled NK1r-immunoreactive neurons were also pyramidal cells (e.g. cells 1 and 2 in Fig. 4).

**Fig. 7:**
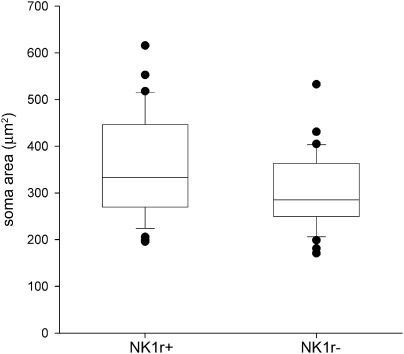
Box and whisker plot of the soma sizes of retrogradely labelled pyramidal cells that were NK1r-immunoreactive (NK1r^+^, *n*=37) or non-immunoreactive (NK1r^−^, *n*=33). The boxes represent the median and interquartile range, while the upper and lower error bars show the 90th and 10th percentiles and the filled symbols are values outside these ranges. Data were taken from the present study and that of Al-Khater and Todd (2009). For further details, see text.

In order to compare the soma sizes of NK1r-immunoreactive and non-immunoreactive pyramidal projection neurons, we pooled the results from the 15 NK1r-negative pyramidal projection cells seen in this study with data from a study of spinoparabrachial neurons in the L3 segment of the rat reported by Al-Khater and Todd (2009). This latter group consisted of 55 retrogradely labelled pyramidal cells, of which 37 were NK1r-immunoreactive and 18 were non-immunoreactive. Soma sizes of the NK1r-immunoreactive pyramidal cells varied from 196 to 616 μm^2^ (median 333, *n*=37) while those of the non-immunoreactive cells ranged from 171 to 533 μm^2^ (median 285, *n*=33). Although the NK1r-immunoreactive cells tended to be larger than the non-immunoreactive ones (Fig. 7), this difference did not reach significance (*P*=0.07, Mann–Whitney *U*-test).

## Discussion

The main finding of this study was that the cell bodies of NK1r-immunoreactive neurons in lamina I had a clear bimodal size distribution that was related to the presence or absence of retrograde labelling from CVLM and/or LPb. The great majority of NK1r-expressing cells that were not retrogradely labelled had soma cross-sectional areas of less than 200 μm^2^, while most of the retrogradely labelled cells were larger than this.

### Choice of injection targets

Lamina I neurons project to several regions of the brainstem and thalamus, however, there is evidence that most or all of the projection cells in this lamina on one side can be retrogradely labelled by injection of tracers into both LPb and CVLM on the contralateral side. Although Ikeda et al. (2006) have provided evidence for a difference in the type of long-term potentiation between lamina I neurons that were retrogradely labelled from PAG or LPb, we have shown that nearly all of the cells that project to the contralateral PAG can also be labelled from contralateral LPb (Spike et al., 2003). This latter observation is consistent with the fact that axons from superficial dorsal horn that travel to the PAG pass through the LPb (Bernard et al., 1995; Feil and Herbert, 1995), since the tracers used by Ikeda et al. and by Spike et al. would have been taken up by fibres of passage, as well as by axon terminals. In addition, we have shown that cells labelled from ipsilateral LPb or CVLM are also labelled from the corresponding sites on the contralateral side (Spike et al., 2003). We have reported that lamina I spinothalamic tract neurons are relatively infrequent in the mid-lumbar segments of the rat (Al-Khater et al., 2008), making up only ∼3–5% of the total number of projection cells in this lamina, and virtually all of these cells send axon collaterals to the LPb (Hylden et al., 1989; Al-Khater and Todd, 2009). Lamina I neurons can also be labelled following injection of tracer into the dorsal part of the caudal medulla (a region that includes the dorsal reticular nucleus and nucleus of the solitary tract; Menétrey and Basbaum, 1987; Lima, 1990; Todd et al., 2000), but we have found that cells labelled from this region are also retrogradely labelled from the LPb (Polgár and Todd, unpublished observations).

Between 83 and 88% of the retrogradely labelled lamina I neurons seen in this study contained Fluorogold and CTb, indicating that they had been labelled from both LPb and CVLM. This proportion is somewhat higher than that reported by Spike et al. (2003), who used similar injection and immunostaining protocols, and found that 63–78% of labelled cells contained both tracers. This difference is mainly due to an increase in the number of cells labelled from the LPb (94–95% of all retrogradely labelled cells in the present study, compared to 81–91% in the study of Spike et al.). Although both studies used antibodies conjugated to Cy5 (which emits mainly outside the visible range) to detect Fluorogold, in the present study this was revealed with a highly sensitive gallium arsenide phosphide (GaAsP) photomultiplier tube, and the resulting improvement in sensitivity probably accounts for the increase in the proportion of cells that were identified as Fluorogold-labelled.

Anterograde tracing studies in cat and monkey (Craig, 1995) have shown the CVLM is a major target for the axons of lamina I projection neurons. However, the CTb injections in this study extended into the ventrolateral white matter, which contains many axons that ascend from the spinal cord (Mehler, 1969; Zemlan et al., 1978). It is therefore likely that some of the retrograde labelling with CTb that was seen in the present study resulted from uptake of the tracer by fibres passing through this region (Spike et al., 2003).

### NK1r-immunoreactive lamina I neurons

The present findings strongly support the hypothesis that the population of large NK1r-immunoreactive neurons identified by Cheunsuang and Morris (2000) consists of projection cells, since virtually all (398/403, 99%) of those with soma areas >200 μm^2^ were retrogradely labelled. In contrast, the size distribution for NK1r-immunoreactive cells that were not retrogradely labelled indicates that these correspond to the population of small neurons. We did not analyse the lateral part of the dorsal horn, because the orientation of lamina I is different in this region, making it difficult to compare soma sizes. However, we have found that retrogradely labelled lamina I neurons in this region have a similar appearance to those elsewhere in the lamina. It is therefore unlikely that that the lack of data from the lateral part of lamina I affects the validity of this conclusion.

The small NK1r-immunoreactive neurons were very numerous and in some cases had very weak immunoreactivity, making them difficult to identify. For these reasons we did not attempt to include all of these cells in the sample that was used for analysis. However, we can be confident that we did not underestimate the extent of retrograde labelling within this group because in all cases a search for retrogradely labelled cells was carried out after the sample of NK1r-immunoreactive neurons had been collected. The absence of retrograde labelling in the small cells is unlikely to be due to lack of time for transport of the tracers, as we have found that increasing post-operative survival time following injection of tracers into the brainstem does not result in any increase in the number of labelled cells or in the appearance of smaller labelled cells (Todd and Polgár, unpublished observations). In addition, it is unlikely to be due to lack of sensitivity of detection for the tracers, since there was a clear distinction between cells that were positive and negative for CTb and Fluorogold. Our findings therefore demonstrate that most of these small cells do not take up tracer that had been injected into LPb or CVLM, Cheunsuang and Morris (2000) speculated that the small NK1r-immunoreactive cells might be projection cells. However, for the reasons stated above, it is very unlikely that these cells project to the thalamus, PAG, LPb, nucleus tractus solitarius, dorsal reticular nucleus or CVLM. Although we cannot rule out the possibility that they project to some other brain region, it is more likely that these cells are intrinsic spinal interneurons.

Three of the NK1r-immunoreactive cells that were not retrogradely labelled had cell bodies >300 μm^2^, and clearly these do not belong to the population of small neurons. Although it is possible that these cells are interneurons, it seems more likely that they are projection cells that were not retrogradely labelled from either of the injection sites used in these studies. The very low number of large (>200 μm^2^) NK1r-immunoreactive cells that were not retrogradely labelled (5/403, 1%) provides additional support for the suggestion that the two injections used in this study labelled the great majority of lamina I projection cells on the contralateral side.

Although we did not quantify the small NK1r-immunoreactive cells, these appeared to be much more numerous than the large ones. This presumably accounts for the relatively high proportion of lamina I cells that are NK1r-immunoreactive (45%; Todd et al., 1998), since projection neurons (∼80% of which express the NK1r) are thought to make up less than 10% of the total neuronal population in this lamina (Bice and Beal, 1997a,b; Spike et al., 2003; Al-Khater et al., 2008).

An important practical outcome from these results is that they can be used to identify putative lamina I NK1r-expressing projection neurons in studies that have not used retrograde tracing. Although NK1r-immunoreactive cells that have soma areas between 150 and 200 μm^2^ cannot be classified with certainty, those with areas above 200 μm^2^ are very likely to be projection cells, while those below 150 μm^2^ are probably interneurons. We have previously shown that a similar approach can be used to identify large NK1r-immunoreactive projection neurons with cell bodies in laminae III or IV and long dorsal dendrites that enter the superficial laminae, since virtually all of the cells of this morphological type were shown to be labelled from the CVLM, with some projecting to LPb and/or the posterior triangular nucleus of the thalamus (Todd et al., 2000; Al-Khater and Todd, 2009).

Little is known about the function of the small NK1r-expressing cells in lamina I. However, since NK1r-immunoreactive cells in this lamina are not GABA-immunoreactive (Littlewood et al., 1995), it is likely that they are glutamatergic excitatory interneurons. We have found that some of these cells phosphorylate extracellular signal-regulated kinases following pinching of the skin, noxious heating or s.c. administration of capsaicin (Al Ghamdi, Polgár and Todd, unpublished observations), which suggests that some, if not all, of them are activated by noxious stimulation. Since they only express low levels of the receptor, it is likely that these cells would not be destroyed by intrathecal administration of substance P conjugated to saporin, and this presumably explains why there is no detectable reduction in the number of neurons in lamina I following this treatment (Nichols et al., 1999).

### Projection neurons that lack the NK1r

This group of cells includes a distinct population of large multipolar neurons, that are characterized by the high density of gephyrin puncta on their cell bodies and dendrites. These cells receive a dense synaptic input from both inhibitory and excitatory interneurons and have been shown to respond to noxious stimuli, since they up-regulate Fos following intraplantar injection of formalin (Puskár et al., 2001; Polgár et al., 2008). However, there are only ∼10 cells of this type on each side in the L4 segment, and they therefore constitute only ∼3% of lamina I projection neurons (Polgár et al., 2008).

Little is known about the function of the remaining NK1r-negative projection neurons in lamina I, although Almarestani et al. (2007) have shown that they include a population of pyramidal neurons that may correspond to the cooling-specific cells identified in the cat (Han et al., 1998). Almarestani et al. (2009) have also provided evidence that these pyramidal cells can display novel expression of the NK1r (and also an increase in innervation by substance P-containing primary afferents) following a chronic inflammatory stimulus. However, there is also a population of large pyramidal NK1r-immunoreactive neurons (Cheunsuang and Morris, 2000) that are included in the spinoparabrachial population (Spike et al., 2003; Al-Khater and Todd, 2009; Almarestani et al., 2007, 2009). It may therefore be difficult to distinguish between pyramidal cells that are normally NK1r-negative and up-regulate the receptor after inflammation, and those that constitutively express the receptor. Almarestani et al. (2009) have reported that the pyramidal cells that normally lack NK1r are smaller than the NK1r-immunoreactive type, and typically have a fourth dendrite that emerges from the dorsal aspect of the soma and travels towards the overlying white matter. However, we found that there was a considerable overlap in the distribution of soma sizes of NK1r-immunoreactive and non-immunoreactive pyramidal neurons, and that the difference between the two populations was not significant. In addition, we only observed a fourth, dorsally-directed dendrite on one of the 15 non-NK1r-immunoreactive pyramidal neurons.

Although cooling-specific projection neurons have been identified in lamina I of the cat and monkey (Craig and Kniffki, 1985; Dostrovsky and Craig, 1996; Han et al., 1998), cells of this type may be rare in the rat, since Bester et al. (2000) did not find cells that responded to innocuous cooling in a sample of 53 lumbar lamina I spinoparabrachial neurons, and although Zhang et al. (2006) identified cervical spinothalamic lamina I cells that were activated by cooling, these cells also responded to noxious stimuli.

## Figures and Tables

**Table 1:** Quantitative retrograde labelling data

Experiment	Retrogradely labelled	Double-labelled	Fluorogold only	CTb only
1	206	182 (88%)	13 (6%)	11 (5%)
2	180	150 (83%)	10 (6%)	20 (11%)
3	156	130 (83%)	8 (5%)	18 (12%)
Total	542	462 (85%)	31 (6%)	49 (9%)

The table shows the total number of retrogradely labelled neurons analysed in each experiment, as well as the number (and percentage) that were double-labelled or labelled only with Fluorogold or CTb.

**Table 2:** NK1r-immunoreactivity in projection neurons

	NK1r-immunoreactivity score					Total
	0	1	2	3	4	
Experiment 1	29 (14%)	56 (27%)	56 (27%)	39 (19%)	26 (13%)	206
Experiment 2	34 (19%)	46 (26%)	35 (19%)	29 (16%)	36 (20%)	180
Experiment 3	38 (24%)	53 (34%)	18 (12%)	24 (15%)	23 (15%)	156
Total	101 (19%)	155 (29%)	109 (20%)	92 (17%)	85 (16%)	542

The numbers (and percentages) of retrogradely labelled cells that were defined as having strong (4), medium (3), weak (2) or very weak (1) NK1r-immunoreactivity, or as being non-immunoreactive (0).
